# Effects of probiotic treatment on the intestinal microbial community of *Haliotis diversicolor*

**DOI:** 10.1186/s13568-025-01885-7

**Published:** 2025-05-31

**Authors:** Ruixuan Wang, Juan Wang, Daguang Tang, Bing Li, Jianjian Huang, Xiaozhi Lin, Yun Li, Wenju Xu, Weifeng Gao, Jiangyong Wang, Hui Zhu

**Affiliations:** 1 Hanshan Normal University, Chaozhou, 521041 China; 2 Guangzhou Jirui Gene Technology Co., Ltd, Guangzhou, 511458 China; 3 Aquatic Product Technology Extension Station, Chenghai District, Shantou, 515824 China; 4 Huizhou University, Huizhou, 516007 China

**Keywords:** *Haliotis diversicolor*, Probiotic, Growth, Microbial community

## Abstract

Probiotic treatment is an effective method for enhancing growth performance and improving intestinal flora in aquaculture species. This study examined the effects of three candidate-probiotics (*Bacillus*, photosynthetic bacteria, and *Lactobacillus*) on the rate of weight gain and the intestinal flora of abalone juveniles. *Haliotis diversicolor* was fed a probiotic-supplemented diet for 30 days. The abalones fed with *Lactobacillus* showed a more significant weight gain rate than those in the *Bacillus*, photosynthetic bacteria, and control groups. Through 16S rRNA high-throughput sequencing, 12,490 amplicon sequence variants (ASVs) were obtained from the abalone intestinal tract microbiome. After a short feeding period (5 days), the *Bacillus* and photosynthetic bacteria-treated groups showed an increased abundance of *Proteobacteria* in the abalone digestive tract. In the *Lactobacillus*-treated group, the quantity of *Proteobacteria* decreased, and the abundance of *Bacteroidot*a increased. After 30 days of feeding, the abundance of *Proteobacteria* and *Bacteroidetes* at the phylum level was more significant in the *Bacillus*-treated group and photosynthetic bacteria-treated group than in the controls. The *Lactobacillus*-treated group showed an increase in the quantity of *Proteobacteria* and Chloroflexi. The dominant flora of the three probiotic treated groups changed slightly with respect to the control group. After a short period of feeding (5 days), the abundance of *Rhodobacteraceae* (at the genus level) in the abalone digestive tract increased in the *Bacillus-* and photosynthetic bacteria-treated groups, whereas in the *Lactobacillus*-treated group, *Rhodobacteraceae* decreased, and *Maribacter* increased in abundance. After 30 days of feeding, *Bacteroidetes* and *Ruegeria* were higher in the *Bacillus*-treated group than in the control group. *Marinirhabdus* and *Bacteroidetes* increased in the photosynthetic bacteria-treated group, and *Roseivivax* and *Ruegeria* increased in the *Lactobacillus*-treated group. The three probiotic-treated groups had higher microbial diversity than the control group. Therefore, our findings confirmed that adding *Bacillus*, photosynthetic bacteria, and *Lactobacillus* to the abalone diet increased abalones’ weight gain rate and altered their intestinal microbiome composition.

**Supplementary Information:**

The online version contains supplementary material available at 10.1186/s13568-025-01885-7.

## Introduction

China is the world’s largest abalone producer. The production of abalones was more than 240 thousand tons in China in 2023, which accounts nearly 90% of the global production (China Fishery Statical Yearbook 2024).Abalone *Haliotis diversicolor* is an essential breeding species in Asia that is distributed in temperate and tropical seas including China, Japan, Korea,Vietnam and other countries in Southeast Asia. And the production of *H. diversicolor* is highly concentrated in south coast of China, mainly in Fujian, Guangdong, Hainan, Guangxi and Taiwan. As one of the most common species of abalone in China (along with *H. discus hannai* and *H. diversicolor*), its output is not published separately, but it is dominant in the southern seas (Fujian, Guangdong, Taiwan, etc.), and is an important variety supporting China’s abalone industry. China contributes nearly 90% of the total global abalone output. Abalone *H. diversicolor*, as an important variety in the main producing areas of south China, plays a key role in the global output growth. 

With intensive aquaculture conditions,high-density feeding results in the rapid accumulation of harmful substances, such as high concentrations of ammonia, nitrogen, and nitrite, in the breeding tanks.These toxic substances lead to the growth of many harmful bacteria and opportunistic pathogens, thereby decreasing the abalone’s resistance to disease and increasing disease incidence (Torrecillas et al. 2007). Traditional disease control methods, such as antibiotics and chemical disinfectants, destroy an organism’s normal flora. Furthermore, they cause an imbalance in the microecology of the organism, thus leading to a decline in its immune function and an increase in drugs-resistant strains (Wang et al. 2014; Wang et al. 2020).

Probiotics are considered beneficial bacteria that can inhibit the growth of pathogenic bacteria and improve the organism’s feed conversion rate (Wang et al. 2020). Probiotics can also regulate intestinal microecology, increase the host’s immune responses, and enhance the host’s resistance to disease (Hai 2015a). More importantly, probiotics influence the host indirectly by affecting the microflora in the digestive tract (Harikrishnan et al. 2011; Liuet al. 2013). In 1989, Kozasa used probiotics for the first time in aquaculture to regulate the intestinal microflora of aquacultured animals from an ecological perspective (Kozasa 1989; Zhao et al. 2018). Probiotics have been confirmed to decrease the concentrations of harmful substances in breeding water and inhibit the reproduction of pathogenic bacteria by maintaining a microecological balance in the digestive tracts of animals(Prol-García and Pintado 2013). Probiotic use is becoming more common in aquaculture, including shellfish and fish because it promotes the growth of cultured species and regulates intestinal microecology (Zhenget al. 2018; Hien et al. 2020; Gaoet al. 2018; Alyssa and Haygood 2018). For example, *Bacillus subtilis*-supplemented diets have been shown to increase the feed efficiency and growth rate of grouper *Epinephelus coioides*, enhance the immune responses of *E. coioides*, and increase resistance to pathogens (Liu et al. 2012).In the past few years, probiotics such as *Streptomyces oligotrophomonas*, *Vibrio*, and *Bacillus* have been isolated from the digestive tract in abalone. *Vibrio* isolated from *H. midae* from South Africa produces an extracellular protease, which was added to the feed to improve the digestion ability of cultured abalone (Huddy and Coyne 2014). Amin et al. have isolated potential probiotics from the digestive tract of *H. laevigata* × *H. rubra*. *Bacillus* showed the highest cellulase activity, which effectively improved the intestinal flora structure and nutritional status (Amin et al. 2016).

However, despite the rapid development of abalone aquaculture treatments with probiotics, the effects of probiotics on abalone culture and the functions of probiotics in the digestive tract remain unclear. Therefore, microorganisms’ complex synergistic and antagonistic interactions and optimal combinations, and the effects of different strains require further study. Hence, to objectively reflect the functions and succession of the intestinal flora in *H. diversicolor*, we analyzed the bacterial communities in *H.diversicolor* after treatment with *Bacillus*, photosynthetic bacteriaand *Lactobacillus* in the present study.

## Materials and methods

### Sources of abalone and probiotics

*H. diversicolor* was collected from Haiyuanda aquaculture Co., LTD in Jieyang City of Guangdong province in China. Juvenile abalones were purchased with an average shell length of 36.7 ± 3.2 mm and a weight of 73.98 ± 6.5 mg. Experimental probiotics were supplied by the Feed and Healthy Aquaculture Technology Development Center, Chinese Academy of Fishery Sciences (Guangzhou, China).Three probiotics including *Bacillus* (trade name: NanShuiLiSheng-01), *Lactobacillus*(trade name: Bioantai-02), and photosynthetic bacteria (trade name: NanShuiLiSheng-02) were added to the plastic tanks. The plastic tanks were then covered with black film for 8 h to activate the probiotics. The total number of *Bacillus* (BA) exceeded 10^9^ cfu/mL, and the total number of photosynthetic bacteria (PB) exceeded 10^8^ cfu/mL. These two probiotics were from Guangzhou Xin Haley Biotechnology Co. Ltd., Guangzhou City, Guangdong, China. The total number of *Lactobacillus* (LA) bacteria exceeded 10^8^ cfu/mL and were from Qingdao Bioantai Biotechnology Co. Ltd., Qingdao City, Shandong, China. The pellet bait was purchased from Weihai gold medal biological technology Co. Ltd., Weihai City, Shandong, China.

### Experimental design

This experiment was performed in 12 plastic tanks, each with a volume of 200 L. After disinfection with a 6 mmol/L potassium permanganate soak, a quadrangle float was added, and filtered seawater was infused. Two pipes were inserted in each tank to ensure adequate dissolved oxygen content. Subsequently, the concretetiles were taken from the abalone pond and moved into the barrel, there were two tiles in every tank. Each tile contained approximately 200 abalones. The types of probiotics added in the feed pellets to each treatment during the abalone culture were BA (base diet *Bacillus*), PB (base diet photosynthetic bacteria), and LA (base diet *Lactobacillus*), respectively. The diet’s final concentration of probiotic bacteria was 5 × 10^7^ cells g^−1^ daily. The control group was fed only the base diet and was labeled C. Feeding was performed daily at 5% wet weight to ensure a suitable environment for abalone growth. Before feeding, *H. diversicolor* feces and bait residues were removed from the culture tank. Three replicates of 200 abalones each were set up. The trial was conducted for 30 days. The water was changed every 7 days, removing 2/3 of the original culture water. Ten abalones (experimental and control groups) were collected from each plastic tank before each water change, and the weights of the abalones were recorded. Throughout the experiment, the water temperature was maintained at 27.8 ± 0.4 °C, the salinity was maintained at 33.2 ± 0.3‰, the pH was maintained at 7.78 ± 0.06, and the dissolved oxygen was maintained at 8.09 ± 0.1 mg/L through continuous aeration.

### Growth performance of abalones

Growth performance was measured on day 30. The weight of 10 abalones from each tank had been respectively measured for theinitial weight at the beginning of the experiment and the final weight after adding probiotics for 30 days. The weight of the abalones in the three replicate tanks for the control and the treated groups were averaged severally, and then the growth rate was respectively calculated and averaged. The rate of weight gain increase was determined as follows:

$$ {\text{Growth rate of weight }}\left( \% \right) \, = \, \left( {{\text{W}}_{{{3}0}} - {\text{W}}_{0} } \right)/{\text{W}}_{0} \times {1}00 $$

W_30_is the average weight of the *H. diversicolor* at the end of the experiment, and W_0_ is the average weight of the *H. diversicolor* at the beginning of the experiment.

### Total DNA extraction

Abalones were collected after 5 and 30 days of rearing and divided into BA_5 (base diet *Bacillus*, treated for 5 d), BA_30 (base diet *Bacillus*, treated for 30 d), PB_5 (base diet photosynthetic bacteria, treated for 5 d), PB_30 (base diet photosynthetic bacteria, treated for 30 d), LA_5 (base diet *Lactobacillus*, treated for 5 d), and LA_30 (base diet *Lactobacillus*, treated for 30 d) groups. Groups C_5 and C_30 were the controls for 5-day and 30-day, respectively. The abalones were washed three times with sterile water to avoid interference from microorganisms on the body surface of the *H. diversicolor* with the digestive tract sampling. The shell was then gently removed with sterile medical scissors. The whole gastrointestinal tract from the stomach to the anus (includingthe hepatopancreas) was then aseptically cut from each abalone with sterile forceps and frozen at − 80 °C until DNA extraction. Genomic DNA was extracted with the bacterial DNA Extraction kit (Manufacturer: Guangzhou Jirui Gene Technology Co., LTD., Guangdong, China). Three replicates of each group were analyzed.

### PCR amplification and pyrophosphate sequencing of 16S rRNA genes

Digestive tract samples were sent to Jirui Gene Technology Co., LTD., (Guangzhou, China) for amplification and sequencing on the Illumina MiSeq sequencing platform. The main operational steps were as follows. The forward primer (338F) 5′-ACTCCTACGGGAGGCAGCA-3′ and reverse primer (806R) 5′-GGACTACHVGGGTWTCTAAT-3′ were used to amplify the V3-V4 region in the 16S rDNA gene. The library was then constructed by “Y” junction ligation, removal of the self-associated fragments through magnetic bead screening, enrichment of the library template with PCR amplification, and denaturing of the library with sodium hydroxide to produce single-stranded DNA fragments. Sequencing was then performed on the Illumina MiSeq sequencing platform. The Illumina sequencing raw data have been submitted to in the NCBI Sequence Read Archive database (accession number: PRJNA1119260).The double-end data were spliced by using overlap, and quality control and chimera filtering were performed to obtain high-quality clean data. Instead of clustering by sequence similarity, the divisive amplicon denoising algorithm (DADA2), which uses steps such as "dereplication" (equivalent to clustering at 100% similarity), was used to obtain single-base representative sequences (Callahan et al. 2016). The core of DADA2 was denoising. Amplicon sequence variants (ASVs) were used to construct the class operational taxonomic units (OTUs) tables to obtain the final ASV feature tables and feature sequences (Blaxter et al. 2005). On the basis of the analysis results for OTUs, α-diversity indexes inculding the Shannon and Chao1 were calculated through random sampling of the sample sequence, and Good's coverage was calculated to evaluate the sampling depth, species taxonomic annotation wereanalyzed with the Bayesian algorithm of the RDP Classifier Ribosomal Database Program,and the bacterial community of each sample was counted at different species classification levels(Tang et al. 2020). Detailed method can be referred to our previous research literature (Xun et al. 2019; Tang et al. 2020; Wang et al. 2020; 2023).

## Results

### Effects of Bacillus, photosynthetic bacteria, and Lactobacillus on the growth performance of H. diversicolor

The weight gain of BA-fed, PB-fed, and LA-fed abalone juveniles were respectively shown in Table 1. The weights of all abalones in the probiotic groups were higher than those in the control group. Among the different probiotic groups, abalones in the LA group gained the most weight (98.5 mg) and weighed substantially more than abalones in the control group (87.8 mg). The PB group followed, with a weight gain of 18 mg and a weight growth rate of 25.17% within 30 days. Abalones in the BA group gained 16.4 mg, with a weight growth rate of 21.27%. *Bacillus*, photosynthetic bacteria, and *Lactobacillus* promoted the growth of juvenile abalones, and *Lactobacillus* had the most pronounced effect on the growth of abalone juveniles. In addition, the survival rate was basically around 99%, and there was no significant difference in survival between the test groups and the control group.

**Table 1:** Effect of dietary probiotics on the weight of juvenile *H. diversicolor*

Parameters	Group C	Group BA	Group PB	Group LA
Initial weight (mg)	74.3	77.1	71.5	73
Final weight (mg)	87.8	93.5	89.5	98.5
Growth rate of weight (%)	18.17	21.27	25.17	34.93

### Composition of the intestinal flora of H. diversicolor and effects of Bacillus, photosynthetic bacteria, and Lactobacillus on flora

In this experiment, 1,442,169 valid sequences were obtained from samples. As shown in Figure S1, the rarefaction curves tended to flatten as the amount of sequencing increased; the rank abundance curve showed a long, flat fold (Figure S1a,b). This result suggested that more data volumes would generate only a small number of new OTUs. The results reflected the microbial diversity of the C group, BA group, PB group, and LA group.

**Fig. 1:**
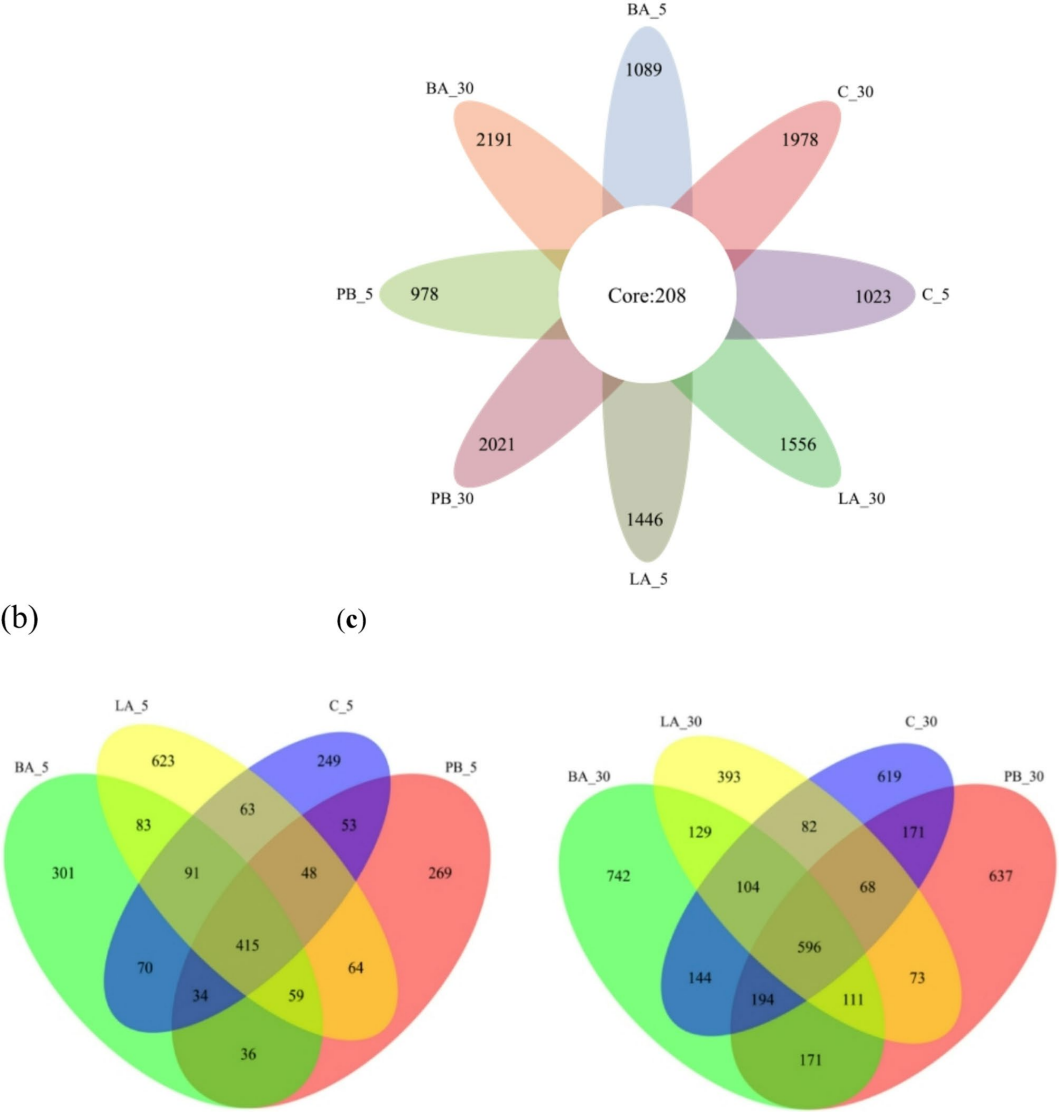
Venn diagrams comparing different groups. *Note*: **a** ASVs are common to each group; **b** ASV distribution in the abalone digestive tract after 5 days of treatment with *Bacillus*, photosynthetic bacteria, and *Lactobacillus*; **c** ASV distribution in the abalone digestive tract after 30 days of treatment with *Bacillus*, photosynthetic bacteria, and *Lactobacillus*. Each circle in the Venn diagram represents a group. The number of overlapping circles represents the number of ASVs common to both groups. The area that does not overlap represents the number of ASVs specific to each group

The distribution of ASVs in the control and experimental groups is shown in Fig. 1. After five days of feeding, the number of ASVs detected in the BA_5 and LA_5 groups were higher, whereas that in the PB_5 group was lower than that in the C_5 group. The number of ASVs in the digestive tract in experimental and control groups increased with feeding duration. After 30 days of feeding with probiotics, the ASV numbers in the BA_30 and BP_30 groups were 2191 and 2021, respectively, which were higher than those in the C_30 group. The ASV number in the LA_30 group was lower than that in the C_30 group. The above results showed that probiotics changed the number of ASVs in the abalones digestive tract, and the number of ASVs increased with feeding duration.

**Fig. 2:**
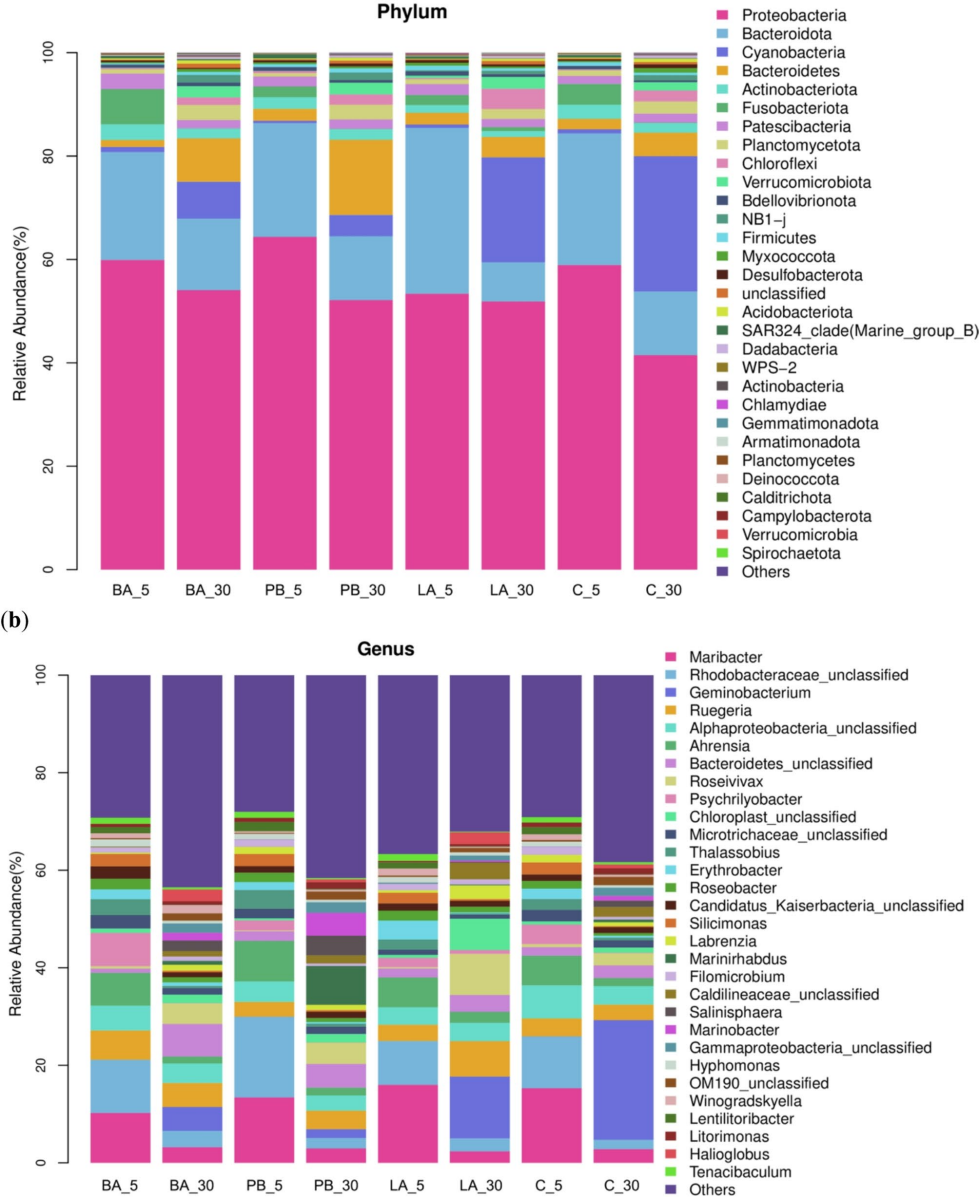
Effects of probiotic-enriched diets on the structural composition and relative abundance of microbial communities in the digestive tract in abalones. *Note*: **a** At the phylum level; **b** At the genus level. The horizontal axis shows the group's name, and the vertical axis represents the relative abundance of a particular taxon; different colors correspond to other species

The composition of the intestinal samples at the phylum level and the effects of the three probiotics on this composition are shown in Figure 2a. The intestinal flora of *H. diversicolor* consisted of primarily ten phyla. Proteobacteria was the dominant group, which accounted for 58.92% of the total sequences. The most common phyla were *Bacteroidota*, *Fusobacteriota*, *Actinobacteriota*and *Bacteroidetes*, accounting for 25.48%, 4.01%, 2.73%, and 2.04% of the total sequences, respectively. The structure of the flora in the digestive tract of *H. diversicolor* was altered after 5 days of feeding on diets supplemented with one of the three probiotics. In the BA_5 group, *Proteobacteria*, *Fusobacteriota*and *Actinobacteriota* were higher, whereas *Bacteroidota*, *Bacteroidetes*and *Firmicutes* were lower than those in the C_5 group. *Proteobacteria*, *Patescibacteria*and *Bacteroidetes* increased, while *Bacteroidota*, *Actinobacteriota*and *Fusobacteriota* decreased in the PB_5 group. The effect of *Lactobacillus* on the composition of the intestinal flora differed from that of *Bacillus* and photosynthetic bacteria. It increased the abundance of *Bacteroidota*, *Bacteroidetes*, *Patescibacteria* and decreased the quantity of *Proteobacteria*, *Fusobacteriota*and *Actinobacteriota* in the LA_5 group. As the feeding time increased, the composition of the bacterial communities in the abalone digestive tract changed. Compared with that in the C_30 group, the abundance of *Proteobacteria*, *Bacteroidetes*and *Bacteroidota* were higher in both BA_30 and PB_30 groups, whereas that of Cyanobacteria was lower. The LA_30 group showed an increase in *Proteobacteria* and *Chloroflexi*, and a decrease in the abundance of *Cyanobacteria* and *Bacteroidota*.

The composition of *H. diversicolor* intestinal samples at the genus level and the effects of the three probiotics on this composition are shown in Fig. 2b. At the genus level, the dominant genus in the digestive tract of *H. diversicolor* was *Maribacter*, accounting for 15.30% of the total number of sequences, and the other dominant genera were *Rhodobacteraceae*unclassified, *Alphaproteobacteria*, and *Ahrensia*, accounting for 10.62%, 6.80%and 6.08%, respectively. *Psychrilyobacter*, *Ruegeria*, *Silicimonas*, and several unknown genera were also present. Compared with that in the C_5 group, in the BA_5 group, the abundance of *Psychrilyobacter*, *Ruegeria*,and *Rhodobacteraceae* was higher, and that *Maribacter* and *Alphaproteobacteria* was lower. In the PB_5 group, the genera that increased in abundance included *Rhodobacteraceae*, *Ahrensia*, and *Thalassobius*, and those that decreased in abundance were *Maribacter*, *Alphaproteobacteria*, and *Ruegeria*. The effects of *Lactobacillus* on the composition of the intestinal flora differed from those of *Bacillus* and photosynthetic bacteria. Increased abundance of *Maribacter*, *Ahrensia*, and *Erythrobacter* in the LA_5 group and decreased abundance of *Rhodobacteraceae*, *Alphaproteobacteria*, and *Ruegeria* were observed. The composition of the bacterial communities in the digestive tract of *H. diversicolor* changed significantly at the genus level with feeding duration. In the BA_30 group, the abundance of *Bacteroidetes*, *Ruegeria*, and *Roseivivax* increased in the digestive tract, while the abundance of *Geminobacterium* decreased. In the PB_30 group, the genera that increased in abundance were *Marinirhabdus*, *Bacteroidetes*, and *Marinobacter*, while *Geminobacterium* decreased. *Lactobacillus* increased the abundance of *Roseivivax*, *Ruegeria*, and *Chloroplast* in the digestive tract and decreased the quantity of *Geminobacterium*.

### Effects of feeding time on the diversity index and composition of the intestinal flora in abalone

**Fig. 3:**
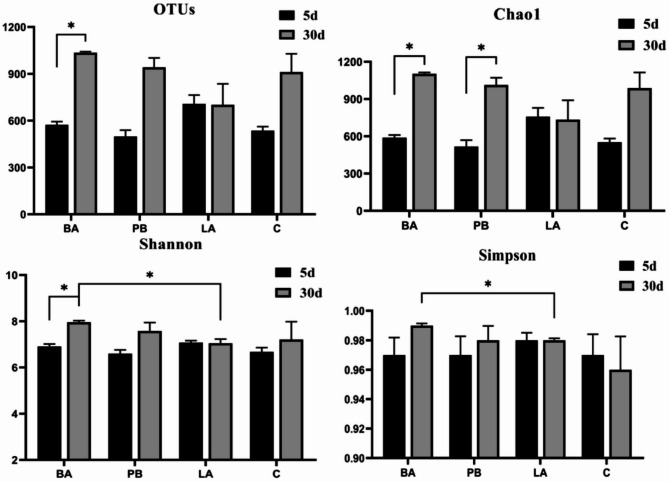
Structural diversity indices of bacterial communities in the digestive tract of *H. diversicolor*. *Note*:*p < 0.05

**Fig. 4:**
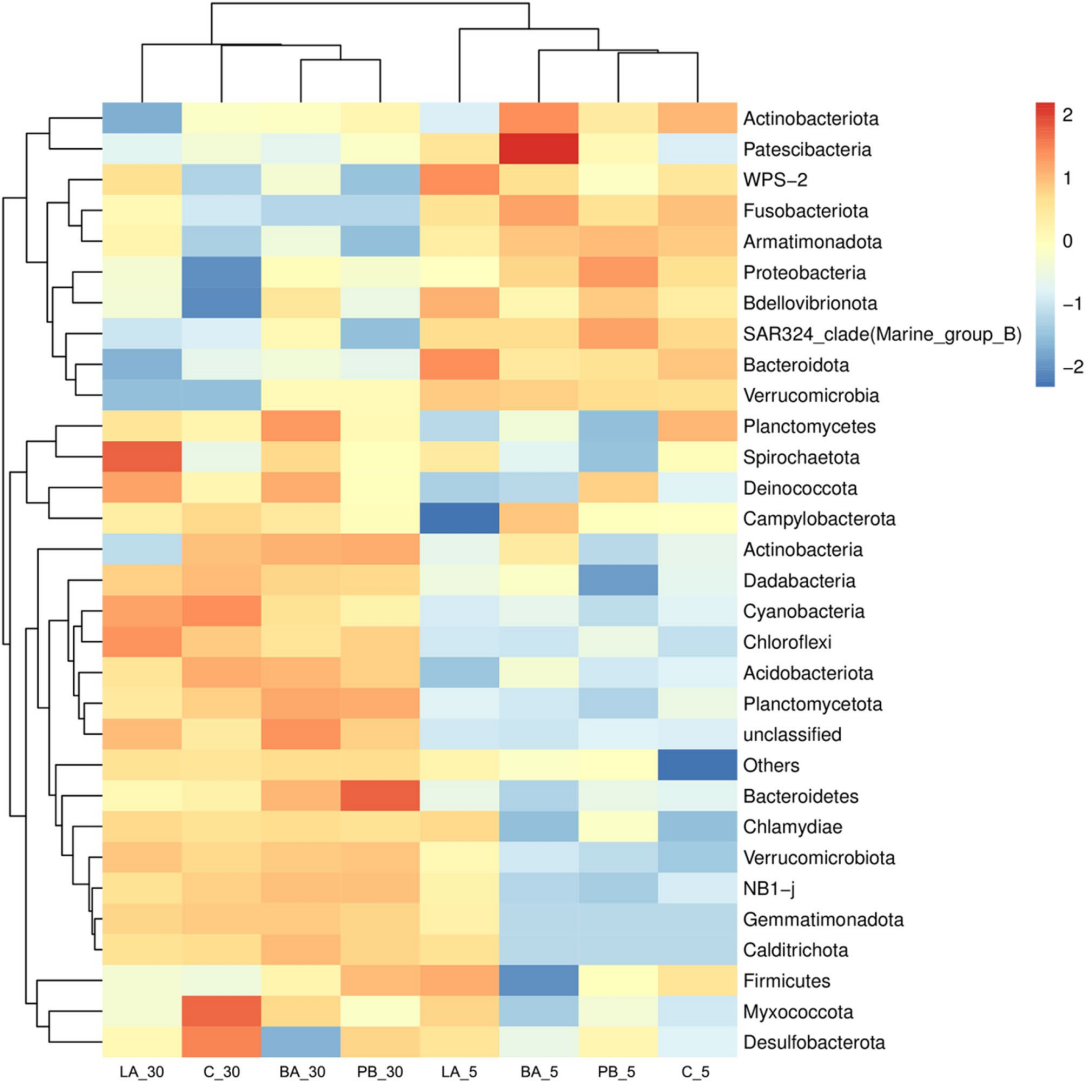
Hierarchical cluster analyses are based on the 30 most abundant OTUs from the intestinal bacterial communities of abalones. *Note*: Each row represents a species, each column represents a group, and the graph reflects the change in abundance from low to high with a gradient from blue to red. The closer to blue, the lower the abundance, and the closer to red, the higher the abundance

The results of the alpha-diversity analysis are shown in Fig. 3. The Chao1 and Shannon indices of BA_5 and LA_5 were higher, while those of PB_5 were lower, than those of the C_5 group. This finding indicated that *Bacillus* and *Lactobacillus* increased the diversity of *H. diversicolor* intestinal flora in a short period (5 d), whereas photosynthetic bacteria decreased the variety of abalone intestinal flora. The Chao1, Shannon, and Simpson indices were higher in the BA_30 and PB_30 groups than in the C_30 group with prolonged feeding time. The Chao1 and Shannon indices were lower in the LA_30 group than in the C_30 group, thus indicating that the diversity of the abalone intestinal flora in the BA_30 and PB_30 groups increased with feeding duration. In contrast, the variety of the abalone intestinal flora in the LA_30 group was slightly lower than that in the C_30 group. In addition, the OTUs and the Chao1 and Shannon indices in the intestinal tract in the abalone in the BA group significantly increased, and the Chao1 index in the PB group increased dramatically as the feeding period was extended from 5 to 30 days (*p* < 0.05). The OTUs, Chao1, Shannon, and Simpson indices for the intestinal tracts of the abalone were approximately the same in the LA_5 and LA_30 groups. This finding suggested that the effects of *Lactobacillus* on the composition of the abalone intestinal flora were less pronounced than those of *Bacillus* and photosynthetic bacteria as the feeding time increased. Figure 4 showed the effects of feeding duration on the composition of the abalone intestinal flora under the same probiotic treatment. The bacterial communities' diversity and abundance in the BA_5 and PB_5 groups did not change significantly with respect to the C_5 group as those in the LA_5 group (Fig. 4). The overall variety and quantity of the bacterial communities in the digestive tract in abalone increased dramatically with longer feeding times (Figs. 3 and 4).In addition, the bacterial communities in the BA_30 and PB_30 groups were similar. As compared with the composition of the digestive tract flora of abalone fed with *Bacillus* and photosynthetic bacteria for 5 days, the abundance of *Fusobacteriota* decreased while the quantity of *Bacteroidete*s, *Cyanobacteria*, *Planctomycetota*and *Chloroflexi* increased after feeding for 30 days (Fig. 4).

### Relationships among the intestinal flora of H. diversicolor after Lactobacillus treatment

**Fig. 5:**
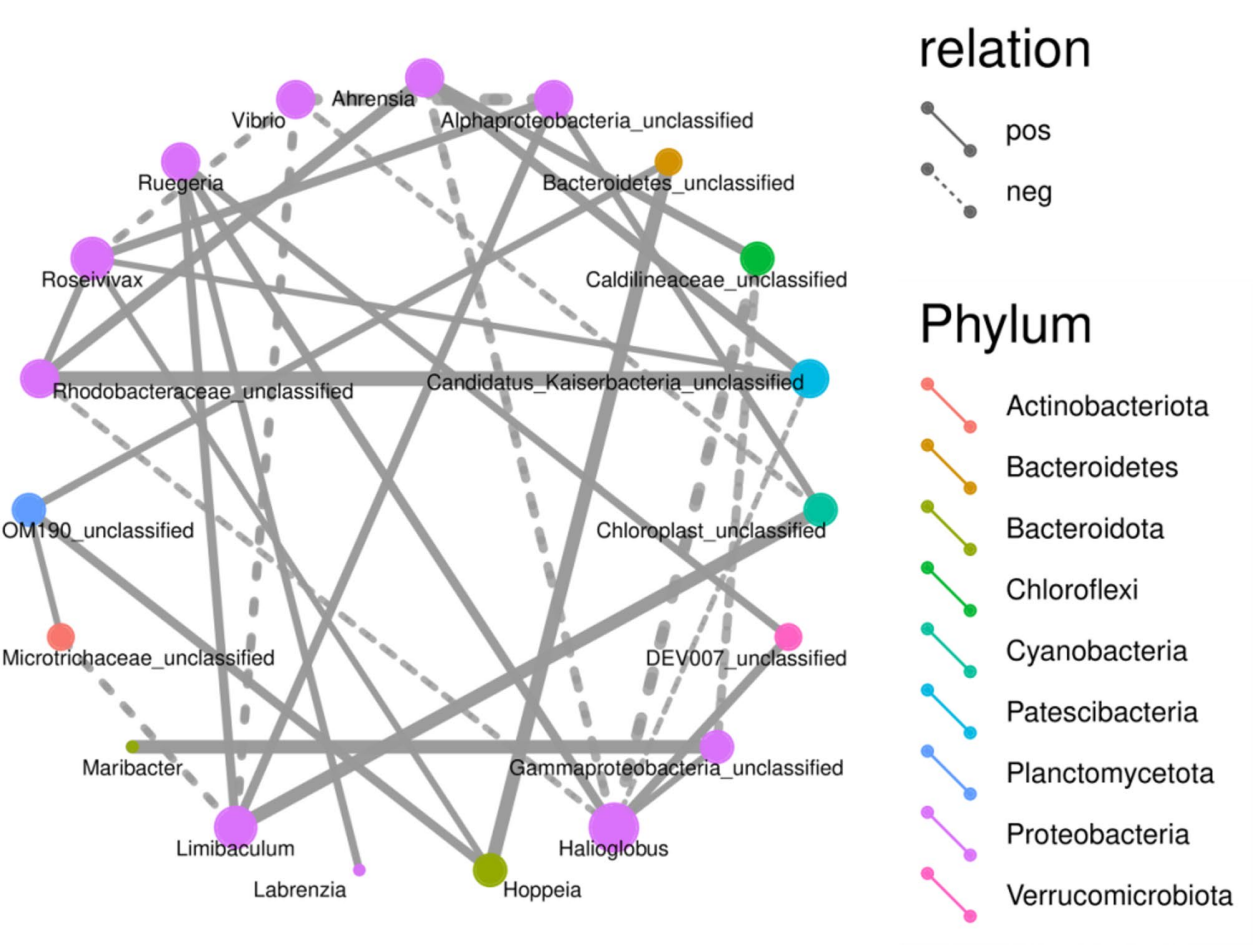
Correlation network diagram for the genus level of the dominant phylum. Note:Different nodes in the network diagram represent other dominant genera, and the node's color indicated the phylum level species to which the species belongs. The connection between nodes means that there is a correlation between two genera, and we show correlation pairs with correlation coefficient|rho|> 0.8 by default, and the thickness of the line indicates the strength of the correlation, with thicker lines indicating stronger correlation and thinner lines indicating weaker correlation. Solid lines indicate positive correlations, and dashed lines indicate negative correlations. The node’s size shows the number of other bacteria associated with the bacteria; the more associations, the larger the node; conversely, the smaller the node

On the basis of the above experimental results, adding *Bacillus*, photosynthetic bacteria, or *Lactobacillus* to the diet clearly increased the rate of weight gain and changed the composition of the flora structure in the abalones. Compared with *Bacillus* and photosynthetic bacteria, *Lactobacillus* had the most significant effect on the growth of the abalones. Figure 4 showed that the intestinal flora structure of abalone treated with *Bacillus* or photosynthetic bacteria was similar, whereas that of abalone treated with *Lactobacillus* differed. To further investigate the effect of *Lactobacillus* on the intestinal flora of abalones and the relationships among flora, we selected the top 20 differentially present genera between LA_30 and C_30 and performed a correlation analysis between genera (Fig. 5). The experimental results indicated a variety of microorganisms belonging to *Proteobacteria*. *Rhodobacteraceae-*unclassified, *Roseivivax*, *Ruegeria*, *Alphaproteobacteria-*unclassified, *Limibaculum*, and *Halioglobus* correlated with one another and other microorganisms. The positive correlations among these species were more significant than the negative correlations. *Rhodobacteraceae-*unclassified had the strongest positive correlation with *Candidatus kaiserbacteria*-unclassified, which belongs to the *Patescibacteria*. A negative correlation with *Halioglobus* was observed, thus suggesting that changes in the abundance of *Rhodobacteraceae-*unclassified were consistent with those in *Candidatuskaiserbacteria*-unclassified after treatment with *Lactobacillus*. The quantity of *Rhodobacteraceae-*unclassified increased, as did *Candidakaiserbacteria*-unclassified, while the amount of *Halioglobus* decreased.

## Discussion

Production and safety in the abalone farming industry are growing concerns. Abalones must be farmed for 3–5 years to reach the marketable size of 80 mm. Therefore, growth performance and survival rates are the most critical factors in the abalone industry. In the past decades, abalone farmers have used antimicrobial compounds and chemicals to prevent disease and increase the growth rate of abalones (Newaj-Fyzul et al. 2014). However, the stress exerted by these chemicals on the environment and the animals has consequences, including bacterial resistance (Wang et al. 2015; Wickramanayake et al. 2020). The use of probiotics as an alternative is an eco-friendly strategy in Chinese aquaculture and has substantially grown in recent years (Hai, 2015b; Liu et al. 2015; Wanget al. 2019).

Verschuere et al. have defined the term “probiotic” as a living microorganism that has a beneficial effect on the host by altering the microbial communities associated with the host, thus ensuring better use of food, enhancing the nutritional value of food and the host's response to disease, or improving environmental quality (Verschuere et al. 2018). In this experiment, *Bacillus*, photosynthetic bacteria, and *Lactobacillus* were added to the daily diet of abalones for 30 days, and the effects of these three probiotics on the weight gain rate and intestinal flora of abalones were investigated. Overall, the study results showed a significantly greater increase in weight gain in *H. diversicolor* fed probiotics than in the control abalones (*H. diversicolor* not fed with probiotics). This result is consistent with findings from previous studies, indicating that the addition of enzyme-producing active probiotics promotes the growth of abalones, including *H. rufescens*, *H. iris*and *H. asinina* L (Silva-Aciares et al. 2011; Hadia et al. 2014; Muhamad et al. 2020). Probiotics' primary mode of action is facilitating the breakdown of nutrients for digestion by the host organism (Wang, 2007). *Lactobacillus*, *Bacillus*and photosynthetic bacteria are rich in exogenous enzymes, such as lipase, protease, amylase, and cellulase, which break down nutrients and promote digestion (Wu et al. 2012; Soltani et al. 2019). *Lactobacillus*, *Bacillus*, and photosynthetic bacteria are sources of many micronutrients, amino acids, short-chain fatty acids, and vitamins beyond digestive enzymes (Pedersenet al. 2005; Nayak 2021; Saejung et al. 2019). Additionally, they can produce a variety of antimicrobial compounds, such as bacteriocins and polymyxins (Ringø et al. 2018; Sharma et al. 2018), thus increasing the resistance of abalones to a variety of pathogenic bacteria (Chopra et al. 2014; Kumar et al. 2016). In the present study, the weight gain rate of *H. diversicolor* increased after feeding the three probiotics, perhaps because of the exact mechanism. Notably, the weight gain rate of abalone supplemented with *Lactobacillus*was higher than that of abalone supplemented with *Bacillus*or photosynthetic bacteria. We speculate that this finding might have been because *Lactobacillus* are anaerobic or partly anaerobic bacteria whose primary metabolite is lactic acid, which can decrease the intestinal pH, and inhibit the reproduction of acid intolerant anaerobic pathogens, and maintain the intestinal micro-ecological balance (Hwanhlem et al. 2010). Moreover, lactic acid increases the digestion and absorption rate, thus promoting the rapid growth of abalone. Photosynthetic bacteria, which are anaerobic or partly anaerobic phototrophs, are not suitable for growth in acidic environments (Zhang 2021). Therefore, the activity of photosynthetic bacteria might be diminished in the abalone digestive tract. Furthermore, *Bacillus* is aerobic or partly anaerobic (Boopathy et al. 2015), and the low oxygen content in the digestive tract may not be conducive to the proliferation of *Bacillus*. Therefore, the rate of weight gain was higher in abalone-fed diets supplemented with *Lactobacillus* than *Bacillus* or photosynthetic bacteria. However, these speculations require further study. In addition, although the weight growth rate of the abalones in the probiotic treatment group was greater than that in the control group, the difference did not reach a significant level (*p* > 0.05). We speculate that this finding might have been due to the short feeding period (30 d). If the feeding period were extended, the three probiotics might have had a more significant effect on the growth of the abalones. Additionally, we also found in our previous research that, *Bacillus* could promote growth and development of *Babylonia areolata*juveniles, and could improve their immunity and disease resistance (Zhang et al. 2023). Thus, in general, probiotics do have a positive effect on aquatic juveniles (Huang et al. 2024).The present study additionally showed that the diversity of the intestinal flora of *H. diversicolor* fed with probiotics was significantly higher. The composition of *H. diversicolor* intestinal flora was altered to that in the control group. The abundance of *Fusobacteriota*, *Patescibacteria*and *Proteobacteria* was higher in the BA_5 group than in the C_5 group. As the feeding time increased, *Proteobacteria*, *Bacteroidetes*and *Bacteroidota* became the dominant bacteria in the digestive tract of *H. diversicolor*. At the genus level, the dominant genus in each probiotic treatment group changed significantly in species composition and abundance as the treatment time was extended from 5 to 30 days. After 30 days of *Bacillus* treatment, the dominant genus in the abalone digestive tract was *Bacteroidetes-*unclassified; in the PB_30 group, the dominant genus was mainly *Marinirhabdus*; and in the LA_30 group, the dominant genus was mainly *Geminobacterium*. However, the dominant species in the *H. diversicolor* digestive tract remained largely stable throughout the culture, with *Proteobacteria* and *Bacteroidota* predominating. This finding might have been due to fluctuations in each taxon in response to dietary changes and the development of their hosts. With longer feeding times, the relationship between host and symbiotic bacteria results from co-evolution between the two entities, as mutually beneficial adaptations that favor their association are selected (Li et al. 2018).

At the phylum level, after treatment with *Bacillus* and photosynthetic bacteria, *Proteobacteria* and *Bacteroidota* were prevalent in the BA and PB groups. They had a higher relative abundance than that in the control group. Previous studies have shown that *Bacillus* increases the quantity of *Proteobacteria* and *Bacteroidota* in pond water (Zhanget al. 2013). Proteobacteria are among the most diverse microbial species on Earth (Huang et al. 2014). *Bacteroidota* is considered the most abundant group of marine bacteria other than *Proteobacteria* and *Cyanobacteria*. It secretes multiple carbohydrate-degrading enzymes and is regarded as the primary utilizer of plant polysaccharides in most intestinal environments. It can support host nutrition by providing fatty acids and vitamins (Larsbrink et al. 2020). After treatment with *Lactobacillus*, the dominant flora in the *H. diversicolor* digestive tract were *Proteobacteria*, *Cyanobacteria*and *Bacteroidota*. No significant changes with respect to the control group were observed, thus suggesting only slight differences in the abalone digestive tract flora composition at the phylum level. Moreover, after treatment, the three probiotics *Bacillus*, photosynthetic bacteria, and *Lactobacillus* were not detected in the *H. diversicolor* intestinal flora. We speculate that this finding might have been because most aquatic animals have a specific digestive tract microbiota established in the juvenile stage, foreign probiotics cannot easily colonize the digestive tract in short periods under normal environmental conditions. This result is also found in our previous analysis of shrimp seedlings (Wang et al. 2020).Therefore, the effective colonization of probiotics and the resulting benefits or rebuilding of the environmental microbial communities must be thoroughly considered (Olafsen 2001; Dawood et al. 2018).

Interestingly, despite being dominant species, *Proteobacteria*, *Cyanobacteria*, *Bacteroidota*and *Bacteroidetes* in the abalone digestive tract showed distinct changes in abundance after 30 days of treatment with the three probiotics. We hypothesize that *Proteobacteria*, *Cyanobacteria*, *Bacteroidota*and *Bacteroidetes* are the core microorganisms in the abalone. The term “core microorganisms” describes taxa with high occupancy rates in a community of animals living in different environments with varying foraging habits (Kokouet al. 2019). Moreover, a strong positive correlation pattern is observed among core microorganisms, in general agreement with the experimental results of the study. We observed significant positive correlations between various genera belonging to Proteobacteria and other microbial species, possibly because of the division of ecological niches, thereby allowing for a low level of competition between these microorganisms and enabling these species to coexist (Costa et al. 2008). We also found negative correlations between some microorganisms, possibly because microorganisms do not exist in isolation but are interconnected with other microorganisms, and face mutual constraints in maintaining a dynamic balance in the abalone intestinal flora. In addition, combined with growth data, our analysis results preliminarily revealed that the changes in abundance of these core flora were beneficial to the growth of the abalone juveniles. Previous research reports have also indicated that introduction of probiotics in the abalone aquaculture could significantly improve the absorption efficiency of nutrients and promote the growth of abalone (Macey et al. 2005; Amin et al. 2020; Muhamad et al. 2020; Moonsamy et al. 2020; Huang et al. 2024).

In summary, the present results suggest that adding probiotics, including *Bacillus*, photosynthetic bacteria, and *Lactobacillus,* to the diet increases the weight gain rate and intestinal flora diversity of *H. diversicolor* and improves the microbial community composition in the *H. diversicolor* digestive tract as feeding time increases. How do probiotics regulate the balance of the bacterial communities? Does *H. diversicolor* show long-term health benefits from this microbial regulation in the digestive tract? Future research could assess these questions through challenge assays, electron microscopy, intestinal immunology, and metagenomic studies.

## Electronic supplementary material

Below is the link to the electronic supplementary material.

Supplementary Material 1.

## Data Availability

The data presented in this study are available in the article.
