# Supplementary material for: Effects of probiotic treatment on the intestinal microbial community of Haliotis diversicolor
Source: AMB Express. 2025 May 31;15:87. doi: 10.1186/s13568-025-01885-7 (PMC12126437; doi:10.1186/s13568-025-01885-7)
Supplement: Supplementary file 1 — Supplementary Material 1. [file 13568_2025_1885_MOESM1_ESM.docx]

(**a**)


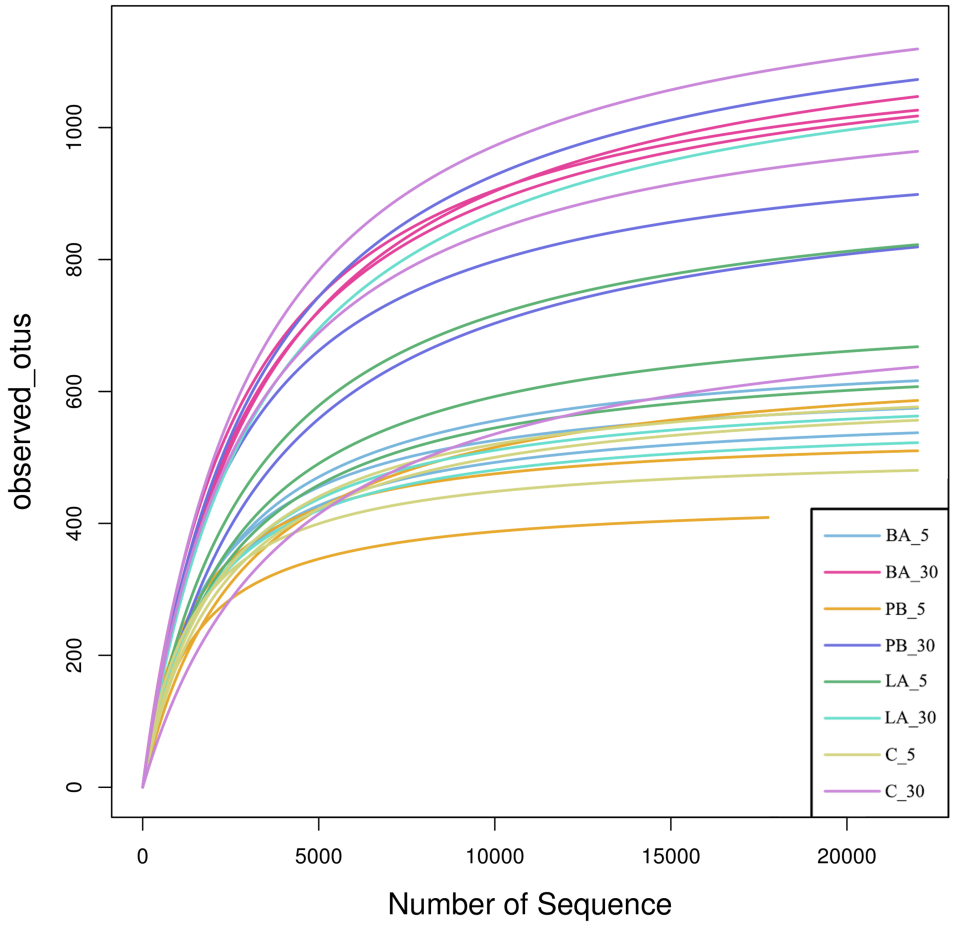


(**b**)


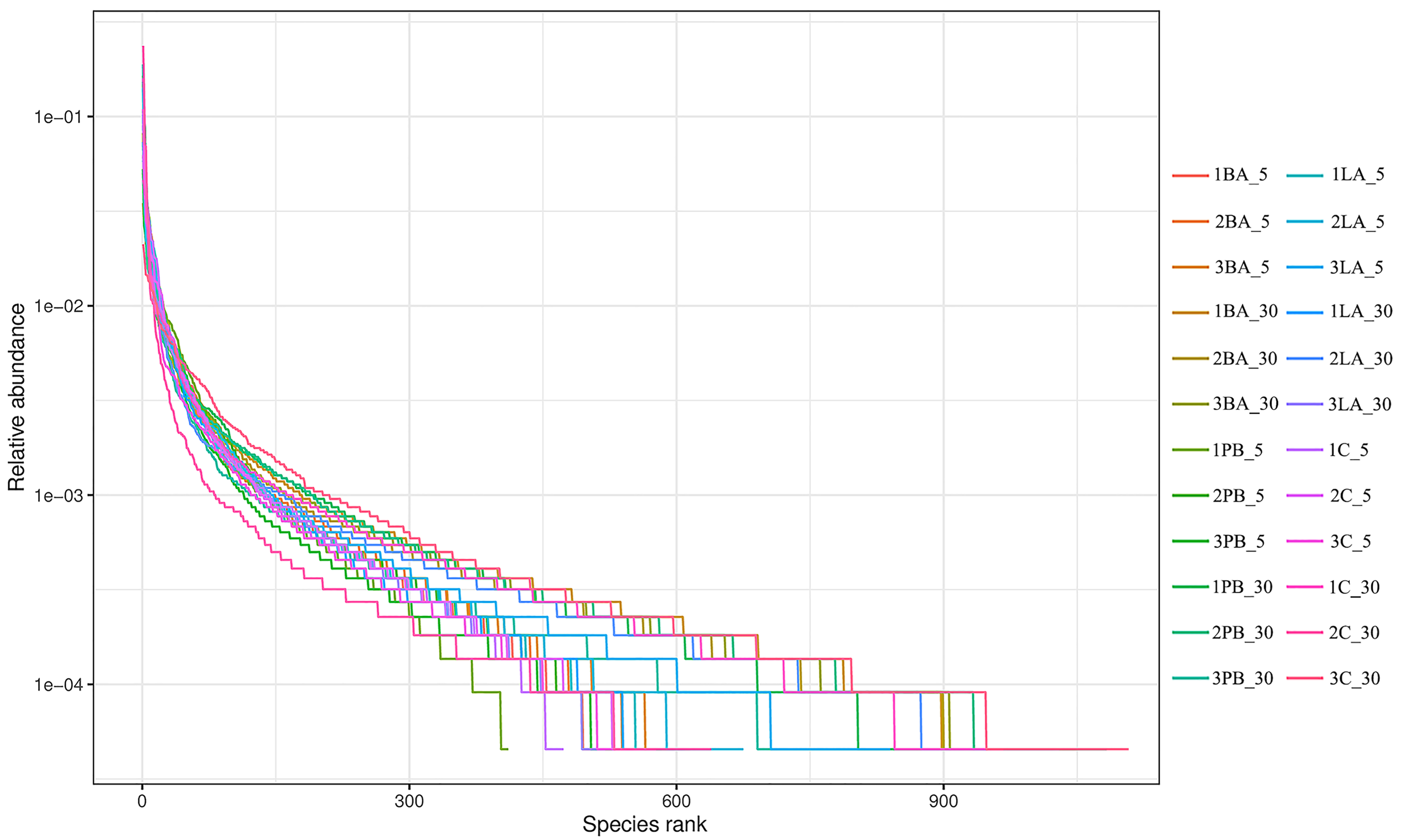


**Figure S1:** The rarefaction curves and rank abundance curve of different groups. (a) The rarefaction curves; (b) rank abundance curve.
